# Assessment of motion and kinematic characteristics of frozen-thawed Sirohi goat semen using computer-assisted semen analysis

**DOI:** 10.14202/vetworld.2015.203-206

**Published:** 2016-02-20

**Authors:** Mukul Anand, Sarvajeet Yadav

**Affiliations:** Department of Veterinary Physiology, College of Veterinary Sciences and Animal Husbandry, Uttar Pradesh Pandit Deen Dayal Upadhayaya Pashu Chikitsa Vigyan Vishwavidyalaya Evam Go Anusandhan Sansthan, Mathura - 281 001, Uttar Pradesh, India

**Keywords:** computer-assisted semen analysis, cryopreservation, motility, semen, Sirohi goat

## Abstract

**Aim:**

The aim was to determine the motion and kinematics characteristic of frozen-thawed spermatozoa in Sirohi goat using computer-assisted semen analysis.

**Materials and Methods:**

A study was carried out in Sirohi buck. Semen collection was made biweekly from each buck with the help of artificial vagina. A total of 12 ejaculates were collected from two bucks (six ejaculates from each buck). Freshly collected semen was pooled and later evaluated. The pooled semen sample was extended with standard glycerolated egg yolk tris extender and later subjected to a process of cryopreservation. The motion and kinematic characteristics of spermatozoa were studied during freez-thawing process.

**Results:**

Significantly (p<0.01) higher value of live percent, hypo-osmotic swelling test, and acrosomal integrity were recorded in neat semen followed by diluted and frozen thaw semen. The proportion of spermatozoa showing slow progression were the highest in the neat and diluted semen followed by rapid and non-progressively motile, while a reverse pattern was observed in the frozen thaw semen where the proportion of non-progressively motile spermatozoa were significantly (p<0.01) higher followed by slow and rapid progression.

**Conclusion:**

This study showed that the best results for motion, vitality, plasma membrane integrity, and acrosome status were obtained in the neat semen followed by diluted and frozen thaw semen. Further, the process of cryopreservation results in a shift of motility from slow to non-progressive in the post-thaw semen with a significant decrease in the path velocities when compared to neat and diluted semen. Hence, it can be concluded that freezing-thawing process reduces the motility and kinematic characters spermatozoa and may be an important factor affecting the fertilizing ability of spermatozoa resulting in poor conception rate after insemination in goats.

## Introduction

Goat has been an integral part of animal husbandry system in India. Due to its peculiar feeding habits and low input cost, the majority of goats are kept and reared by small, marginal and landless farmers in country [1]. But with the increase in population and decrease in the pasture land, goat husbandry practice is gaining importance as an alternate food source in our country. There has been a tremendous increase in number of commercial goats farms in recent years [2]. However, due to indiscriminate breeding and unawareness among the animal owners, the productive and reproductive efficiency of goats lags behind and need improvement. Establishment of true line breeds with high proliferacy and fast growth are major challenges to be addressed. Selective breeding of goats can be alternate for genetic upgradation. Artificial insemination (AI) through the use of cryopreserved semen is an important tool for successful implementation of breeding program to establish pure line breed of goat [3]. The success of AI largely depends on quality of cryopreserved semen that in turns determines the conception rate [4]. Hence, quality assurance through regular monitoring of semen quality has becomes an important component of freez-thawing protocol. Different parameters have been establish for assessment of semen quality, *viz*., percent live, hypo-osmotic swelling test (HOST), acrosomal integrity, motility, etc. that defines the quality of semen.

Motility is a phenomenon responsible for transport of spermatozoa from site of deposition to site of fertilization. This process peculiar to spermatozoa depends on the physiological and morphological status of a sperm cell and is highly sensitive to stressor encountered during the process of cryopreservation. Earlier the motility was evaluated through visual observation, i.e., mass motility and progressive motility that lack precision. Further, motility characteristic is considered as the parameter of choice to determine the degree of sperm damage inflicted by the cryopreservation in goats due to the wide range of results found for sperm motility [5].

Hence, taking into account the importance of motility and motion characters exhibited by sperms in determining the fertility, computer-assisted semen analysis (CASA) techniques is now being used to study motion and path velocities of spermatozoa. CASA provides an objective and reproducible data on a number of sperm motion parameters and enhance the value of motility assessment to fertility prognosis resulting in high correlations among several CASA motility parameters [6,7]. Since the identification of ejaculates as suitable, or not suitable is based on fresh and post-thaw semen quality [8], the study was conducted to evaluate the motility and kinematic parameters in the neat, diluted and frozen thaw semen of Sirohi buck.

## Materials and Methods

### Ethical approval

No ethical approval was necessary to pursue this research work.

### Animal and sampling

The study was conducted on 2 Sirohi bucks aged between 3 and 4 years, weighing between 30 and 40 kg, reared in experimental goat shed of Department of Physiology, Pt. Deen Dayal Upadhaya Pashuchikitisha Vigyan Vishwa Vidyalay Evam Go Anusandhan Sansthan (DUVASU), Mathura. The work related to semen analysis was performed in the Department of Veterinary Physiology (Hi-Tech Lab), College of Veterinary Science and Animal Husbandry, DUVASU, Mathura, Uttar Pradesh Semen collection was made biweekly from each buck with the help of artificial vagina. A total of 12 ejaculates were collected from two bucks (six ejaculates from each buck). Freshly collected semen was evaluated and pooled. The pooled semen (spermatozoa with seminal plasma) was extended with standard glycerolated egg yolk tris extender (3% egg yolk and 6% glycerol) and later subjected to process of cryopreservation. Sperm attributes *viz*., percent live sperms [9], HOST [10] and sperms with intact acrosome [11] were evaluates manually using standard protocol while sperm kinematics were evaluated using the CASA system during the free-thawing process. To assess post-thaw sperm parameters, straws were removed from liquid nitrogen storage container with the help of forceps and dipped in water maintained at 37°C for 30 s in thawing unit (IMV, France). Later straws were withdrawn and wiped with tissue paper to make it moisture free. Sperm motion parameters and sperm kinematics were evaluated using CASA on a plate warmed to 37°C, negative phase contrast and ×10 objective. Settings of the CASA system (Biovis CASA 2000, Version 4.6, India) designed using algorithm based on size, shape, detection of sperm head and classes for motile, immotile, rapid, slow and non-progressive were as follow: Frames/s - 60, number of frames acquired - 61, max velocity (for tracking): V (um/s) - 150 motility min, curvilinear velocity (VCL): V (um/s) - >25 motility min, average path velocity: V (um/s) - >10 motility min, straight-line velocity: V(um/s) - >1 min, track length (% of frames) - 51, aspect - 0-99,999, area - 2-20, axis (major) - 4-20, axis (minor) - 2-10, compactness - 0-50, perimeter ratio - 0-99,999, minimum cell size on major axis - 20, minimum cell size on min axis - 10 magnification - ×10 phase, calibration × (pixels/unit) - 1.905 μ, Y (pixels/unit) - 1.905 μ, size of image - 1280 × 960 pixels. Semen was diluted and adjusted to a concentration of about 50 × 10^6^ spermatozoa per ml for computer aided motility analysis. A 4 μl of diluted semen sample was loaded in metallic sperm counting chamber and a range of 3-6 fields were acquired for motility analysis.

### Statistical analysis

Statistical analyses were performed using Statistical Package for Social Science (SPSS^®^ Version 20.0 for Windows^®^, SPSS Inc., Chicago, USA). The means were compared using Analysis of Variance, Duncan's multiple range test and presented as mean ± standard error (SE).

## Results and Discussion

**Figure-1:**
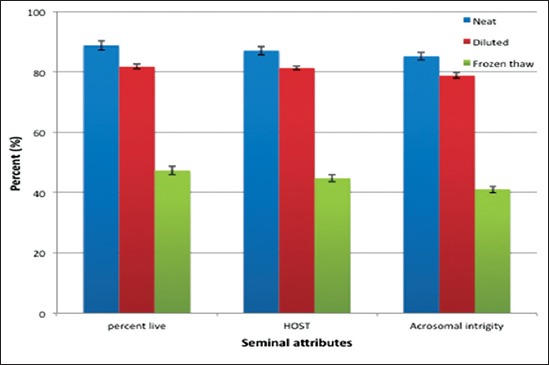
Different seminal attributes in Sirohi goat subjected to freezing-thawing process. Significance level (p<0.01).

The effects of freeze-thawing process on live percent, HOST and percent sperms with intact acrosome has been shown in Figure-1. The observed mean (±SE) values different seminal attributes were well within the normal range at all the three steps evaluated during the experiment. A significant difference (p<0.01) was observed in all the three steps studied during the freez-thawing process with highest values observed in the neat followed by dilution and post-thaw semen. Significantly (p<0.01) lower value recorded for diluted semen compared to the neat may be the result of lethal interaction between the seminal plasma and egg yolk in semen extender [12] supplemented with osmotic disturbance. Buck seminal plasma contains an enzyme secreted by the bulbo-urethral glands, which in the presence of egg yolk, by hydrolysis, leads to the formation of lysophosphatidylcholines – which are toxic to sperm [12,13]. Exposure of sperm to extracellular solute concentration leads to transport of water and solutes, including ions into or out of the cell, that affects the sperm osmotic tolerance limits above and below affecting motility and viability of spermatozoa [14]. Significantly (p<0.01) lower values recorded in the frozen thaw semen compared to neat and diluted semen may be the result of cryogenic stress incurred by spermatozoa during freezing-thawing process. During the process of semen cryopreservation spermatozoa are subjected cooling, freezing-thawing, and the addition of cryoprotectants. Spermatozoa subjected to cryopreservation are very sensitive to a rapid reduction in temperature from room temperature to 5°C [15] this produces cold shock, freezing-induced dehydration of the cells causes a more severe phase transition to a highly ordered gel phase resulting in a loss of selective permeability and integrity of the plasma membrane [16] leading to loss of motility and diminished metabolism.

Different motility patterns exhibited by spermatozoa during the freez-thawing process have been presented in Table-1. Significantly (p<0.01) higher number of motile spermatozoa was observed in the neat semen followed by diluted and frozen thaw semen. The proportion of spermatozoa showing slow progression were significantly (p<0.01) higher in the neat and diluted semen followed by rapid and non-progressive, while a reverse pattern was observed in the frozen thaw semen where the proportion of spermatozoa with non-progressive motility were significantly (p<0.01) higher followed by slow and rapid progression. The difference in the motility pattern exhibited by the spermatozoa may be the result of semen dilution resulting in hypertonic stress during the addition of cryoprotective agents [17], effect of egg yolk [18], disturbed plasma membrane integrity and mitochondrial activity that influenced the sperm morphology and physiology after dilution. Further, freezing-thawing result in overproduction of reactive oxygen species (ROS) [19]. ROS affects the membrane structure, leads to a change in permeability and probably influence integrity of cellular organelles (e.g. mitochondria, endoplasmic reticulum, etc.), impairs cellular structure and destroys membranes by interacting with biomolecules that affects the viability and motility of spermatozoa and can also induce oxidative stress [20].

**Table-1:** Motility pattern exhibited by spermatozoa during freezing-thawing process.

Parameter (%)	Neat semen	Diluted semen (tris-glycerol)	Frozen thaw semen
Motility	83.80^c^±0.949	79.15^b^±0.625	41.70^a^±0.819
Rapid	16.87^b^±0.970	13.40^b^±1.434	1.10^a^±0.331
Slow	57.00^b^±0.544	56.10^b^±0.821	12.12^a^±1.205
Non- progressive	9.92^b^±0.975	9.70^b^±0.481	28.42^a^±1.647

Means with different superscript letters (a, b, c) differ significantly (p<0.01) within a row

The different path velocities and kinematic characters exhibited by spermatozoa at different time interval have been presented in Table-2. Non-significantly higher values were recorded for all the path velocities except for VCL which was significantly (p<0.01) higher in the neat semen as compared to diluted semen. This difference in the velocity patterns of spermatozoa may be the result of reduced tolerance to hypotonic stress, a decreased intracellular pH and increased intracellular potassium affecting mitochondrial function and cell physiology [21]. Significantly (p<0.01) lower values of different path velocities were observed in post-thaw semen when compared to the neat and diluted semen. The results recorded during the study are comparable with those observed by Amidi *et al.*, [22] and Bezerra *et al.*, [23] in cryopreserved goat semen. The spermatozoa during the process of freez-thawing undergo stress. This leads to production of ROS, movement of proteins and cholesterol leading to change in membrane permeability accompanied by osmotic stress and ice crystal formation. The formation of intracellular ice crystals and ROS production result in the concentration of solutes remaining in the unfrozen fraction increases, thereby both depressing the freezing point and increasing the osmotic pressure altering sperm function [24,25]. Induction of premature acrosomal reaction, altered mitochondrial function and failure of chromatin decondensation, during the process of cryopreservation which influences the motility, viability and fertility of the sperm cells [26].

**Table-2:** Different path velocities exhibited by spermatozoa during freeing-thawing process.

Parameter	Units	Neat semen	Diluted semen (tris-glycerol)	Frozen thaw semen
VCL	μm/s	102.00^a^±1.08	96.75^b^±1.10	63.75^c^±0.47
VAP	μm/s	42.25^b^±0.478	41.00^b^±0.707	30.35^a^±0.478
VSL	μm/s	35.75^b^±0.629	34.50^b^±0.645	18.50^a^±0.288
LIN	%	34.95^b^±0.184	35.72^b^±0.221	29.60^a^±0.470
STR	%	83.37^b^±0.449	83.07^b^±0.469	61.62^a^±1.291
WOB	%	41.62^a^±0.209	42.60^a^±0.168	47.65^b^±0.518
BCF	hz	19.00^b^±0.108	18.25^b^±0.366	12.65^a^±0.087
ALH	μm	5.15^b^±0.028	5.07^b^±0.062	3.57^a^±0.047
DNC	μm²/sec	422.00^b^±8.348	393.00^b^±5.922	173.77^a^±0.667

Means with different superscript letters (a, b, c) differ significantly (p<0.01) within a row. VLC=Curvilinear velocity, VAP=Average path velocity, VSL=Straight line velocity, LIN=Linearity, STR=Straightness, WOB=Wobble, BCF=Beat cross frequency, ALH=Amplitude-lateral head displacement, DNC=Dance

## Conclusion

From the findings of the present study, it can be concluded process of cryopreservation results in shift of motility pattern of spermatozoa from slow progression to non-progression in the post-thaw semen with a significant (p<0.01) decrease in the path velocities when compared to neat and diluted semen. Further, the freezing-thawing process that reduces the motility and kinematic characters spermatozoa may be an important factor influencing the fertilizing ability of spermatozoa resulting in poor conception rate after insemination in goats.

## Authors’ Contributions

SV and MA designed and conducted the study. MA drafted and revised the manuscript. Both authors read and approved the final manuscript.
